# CHARGE-AF in a national routine primary care electronic health records database in the Netherlands: validation for 5-year risk of atrial fibrillation and implications for patient selection in atrial fibrillation screening

**DOI:** 10.1136/openhrt-2020-001459

**Published:** 2021-01-18

**Authors:** Jelle C L Himmelreich, Wim A M Lucassen, Ralf E Harskamp, Claire Aussems, Henk C P M van Weert, Mark M J Nielen

**Affiliations:** 1 Amsterdam UMC, University of Amsterdam, Department of General Practice, Amsterdam Public Health, Amsterdam, The Netherlands; 2 Netherlands Institute for Health Services Research, Utrecht, The Netherlands

**Keywords:** atrial fibrillation, risk factors, epidemiology, electronic health records

## Abstract

**Aims:**

To validate a multivariable risk prediction model (Cohorts for Heart and Aging Research in Genomic Epidemiology model for atrial fibrillation (CHARGE-AF)) for 5-year risk of atrial fibrillation (AF) in routinely collected primary care data and to assess CHARGE-AF’s potential for automated, low-cost selection of patients at high risk for AF based on routine primary care data.

**Methods:**

We included patients aged ≥40 years, free of AF and with complete CHARGE-AF variables at baseline, 1 January 2014, in a representative, nationwide routine primary care database in the Netherlands (Nivel-PCD). We validated CHARGE-AF for 5-year observed AF incidence using the C-statistic for discrimination, and calibration plot and stratified Kaplan-Meier plot for calibration. We compared CHARGE-AF with other predictors and assessed implications of using different CHARGE-AF cut-offs to select high-risk patients.

**Results:**

Among 111 475 patients free of AF and with complete CHARGE-AF variables at baseline (17.2% of all patients aged ≥40 years and free of AF), mean age was 65.5 years, and 53% were female. Complete CHARGE-AF cases were older and had higher AF incidence and cardiovascular comorbidity rate than incomplete cases. There were 5264 (4.7%) new AF cases during 5-year follow-up among complete cases. CHARGE-AF’s C-statistic for new AF was 0.74 (95% CI 0.73 to 0.74). The calibration plot showed slight risk underestimation in low-risk deciles and overestimation of absolute AF risk in those with highest predicted risk. The Kaplan-Meier plot with categories <2.5%, 2.5%–5% and >5% predicted 5-year risk was highly accurate. CHARGE-AF outperformed CHA_2_DS_2_-VASc (Cardiac failure or dysfunction, Hypertension, Age >=75 [Doubled], Diabetes, Stroke [Doubled]-Vascular disease, Age 65-74, and Sex category [Female]) and age alone as predictors for AF. Dichotomisation at cut-offs of 2.5%, 5% and 10% baseline CHARGE-AF risk all showed merits for patient selection in AF screening efforts.

**Conclusion:**

In patients with complete baseline CHARGE-AF data through routine Dutch primary care, CHARGE-AF accurately assessed AF risk among older primary care patients, outperformed both CHA_2_DS_2_-VASc and age alone as predictors for AF and showed potential for automated, low-cost patient selection in AF screening.

**Key questions**

**What is already known about this subject?**

- Patient selection in atrial fibrillation (AF) screening studies has so far been based mainly on high age. There are indications, however, that multivariable risk prediction models are better at discriminating for high and low risk of AF in the community than age alone. A recent systematic review and meta-analysis showed that Cohorts for Heart and Aging Research in Genomic Epidemiology model for atrial fibrillation (CHARGE-AF) may be the best suitable risk model for this purpose in community cohorts.

**What does this study add?**

- Previous validations of CHARGE-AF have been performed mainly in prospective community cohorts with high completeness of data. If the model were to be used for low-cost, automated patient selection in AF screening, however, it is more likely that researchers will turn to readily available routine primary care data, without a costly baseline visit for each eligible patient. This study is the first to provide detailed information on how selecting at different cut-offs of CHARGE-AF risk would translate into numbers of patients to be screened and percentage of AF yield to be expected while using a large European routine primary care dataset.

**Key questions**

**How might this impact on clinical practice?**

- Outcomes of this work are relevant to the prospect of using clinical risk models as triage test for AF screening, while also maintaining low cost in their risk assessment efforts. We showed that those with complete CHARGE-AF variables as per routine primary care constitute a small but highly relevant subset for AF screening. CHARGE-AF’s high accuracy in predicting absolute 5-year year risk for predefined risk categories suggests that the model can be used to reliably differentiate between low and high AF risk among cases with complete CHARGE-AF data through routine primary care. Moreover, CHARGE-AF can do so with higher accuracy than two predictors that are currently used as triage tests for AF screening: age alone and the congestive heart failure, hypertension, age, diabetes and previous stroke or transient ischaemic attack, vascular disease and female sex categoryCHA2DS2-VASc score. This work therefore encourages researchers in the field of community AF screening to consider CHARGE-AF as a triage test for patient selection.

## Introduction

Atrial fibrillation (AF) is a common arrhythmia increasing in incidence with age.^1^ It is associated with a higher risk of ischaemic stroke for which effective prophylactic treatment is available.^2^ There is increasing interest in more efficient strategies for early AF detection in the ageing community.^3^ One approach is the use of multivariable risk models for patient selection in AF screening: longer or more frequent follow-up in patients with higher risk and less stringent regimes in the lower risk strata.^4^

The Cohorts for Heart and Aging Research in Genomic Epidemiology model for atrial fibrillation (CHARGE-AF) model predicts an individual’s 5-year risk of new AF using relatively easily obtainable variables: age, ethnicity, height, weight, systolic blood pressure (SBP), diastolic blood pressure (DBP), current smoking, antihypertensive medication use, diabetes mellitus (DM), heart failure and myocardial infarction (MI).^5^ CHARGE-AF was derived and calibrated in community-dwelling older subjects of European and African descent. It has been validated in various community cohorts^5–10^ and appears to be the most viable prediction model for patient selection in future community AF screening.^11^

To further increase efficiency of risk model-assisted AF screening efforts, minimal resources should be required to adequately perform baseline risk stratification.^3^ One eligible data source for this purpose are primary care electronic health records (EHRs). However, while age and cardiovascular morbidities can be deduced from primary care EHRs with high completeness, other CHARGE-AF variables may not be as frequently recorded. Most notably, the body measurements required in CHARGE-AF—height, weight, SBP and DBP—have been shown to often be incomplete in real-world primary care data, with selective reporting favouring those with higher comorbidity rates.^12 13^

If CHARGE-AF were shown to be a valid risk stratification tool within the subset of patients with readily available complete data for CHARGE-AF risk assessment, and if this subset were to constitute a population with clinical significance for AF screening, this could point to a reduced necessity for a baseline visit prior to risk stratification in these patients. We therefore set out to perform a retrospective cohort study using a nationwide primary care EHR database with three aims:

1. To study the subgroup of primary care patients with recent and complete baseline data for the CHARGE-AF variables in terms of relevance for AF screening.
2. To validate CHARGE-AF for 5-year AF risk and to compare it with other established predictors for AF in complete CHARGE-AF cases.
3. To explore how a choice of baseline CHARGE-AF risk cut-offs could affect patient selection and potential AF yield in future AF screening among complete CHARGE-AF cases.

## Methods

We reported this study in accordance with the Transparent Reporting of a Multivariable Prediction Model for Individual Prognosis or Diagnosis statement.^14^

### Netherlands Institute for Health Services Research Primary Care Database (Nivel-PCD)

The Nivel-PCD consists of routine primary care EHR data from over 1.8 million patients from over 500 general practices across the Netherlands in 2019. The database includes information on diagnoses, consultations, prescribed medication and (laboratory) measurements.

In the Netherlands, all non-institutionalised inhabitants are obligatorily registered with one general practitioner (GP) as their primary care provider. In general practices, all encounters are linked to International Classification of Primary Care version 1 (ICPC-1) diagnostic codes in the EHR.^15^ Since GPs have a central role in Dutch primary care as the gatekeepers of referrals to specialised care, all specialists report their findings back to the GP. The GP then links this correspondence to either an existing or a new ICPC-1 code. Therefore, GPs have a complete overview of morbidity of their patients. Nivel-PCD constructs episodes of illness with associated start and end date using multiple markers of diagnostic information in the EHRs (see online supplemental methods for details). This process has been described previously and has been shown to provide an accurate assessment of morbidity rates.^16^

10.1136/openhrt-2020-001459.supp1 Supplementary data

Prescriptions are recorded according to the Anatomical Therapeutic Chemical classification system. Since GPs in the Netherlands are often tasked with providing repeat prescriptions for medication initiated by specialists, Nivel-PCD widely covers prescriptions for chronic morbidities initiated by both GPs and specialists. Other data including but not limited to sex, age, smoking status and body measurements are stored as separate parameters. Due to prohibitions by Dutch law, information on ethnic background is not systematically recorded in EHRs.^17^

### Data extraction

We used data from 1 January 2013 to 31 December 2018. Baseline was 1 January 2014, with the EHR data recorded during the calendar year 2013 serving as baseline data in order to include only recent measurement and medication data. When multiple entries for one variable were available in 2013, we used the recorded entry closest to baseline, 1 January 2014. Detailed operational definitions for the CHARGE-AF variables are shown in the online supplemental methods.

We assumed absence of baseline morbidity or smoking when no episode of illness or status as active smoker was recorded for a disease prior to baseline.^18^ Age and sex were available for all patients. When a patient had no recorded height, weight, SBP or DBP during calendar year 2013, we considered these measurements as missing. We applied no imputation techniques for missing CHARGE-AF measurement variables since we expected these data not to be missing at random.

### Study population

We included patients aged 40 years or older and free of AF at baseline who were registered at one of the Nivel-PCD associated practices during the full calendar year 2013. We excluded patients from practices without follow-up data beyond 2013 since inclusions of such data would automatically render patients without follow-up data. Among included patients, we distinguished those with missing data for one or more of the four body measurements included in the CHARGE-AF model (height, weight, SBP and DBP)—‘incomplete cases’—and those with baseline data available for all these measurements—‘complete cases’.

### Outcomes

The primary outcome was newly diagnosed AF. We defined AF as the recording of the ICPC-1 code K78 ‘AF or atrial flutter’ or any recording of a treating physician for AF or participation in AF care programme. We defined the date of AF diagnosis as the first date associated with either of these AF entries. We were unable to ascertain death as the reason for loss of follow-up, since date and cause of death are not validly recorded in primary care EHRs.

### Follow-up

Patient registration at a Nivel-PCD associated practice is assessed quarterly. Reasons for loss of follow-up in Nivel-PCD are death, exclusion of practice due to low quality data, technical failure of data extraction or a patient moving away from their Nivel-PCD associated practice. We defined loss to follow-up as the first day of a period of four or more consecutive quarters of absent data, or the first day of a period of consecutive quarters of absent data that included the last quarter of calendar year 2018. We censored follow-up in our analyses at time of AF diagnosis, loss to follow-up or end of the 5-year observation window (31 December 2018), whichever occurred first.

### The CHARGE-AF model

We calculated each individual’s CHARGE-AF predicted 5-year AF risk using the formula from the original derivation article^5^: 1–0.9718412736 ∧ exp (ΣbX − 12.5815600). Here, ΣbX is calculated as: (age in years/5) * 0.5083+ethnicity (Caucasian/white) * 0.46491 + (height in centimetres/10) * 0.2478 + (weight in kg/15) * 0.1155 + (SBP in mm Hg/20) * 0.1972 – (DBP in mm Hg/10) * 0.1013+current smoking * 0.35931+antihypertensive medication use * 0.34889+DM * 0.23666+heart failure * 0.70127+MI * 0.49659.

The Dutch population is ~95% Caucasian/white,^19^ and Nivel-PCD contains a representative sample of Dutch inhabitants.^20^ In absence of ethnicity data in Nivel-PCD, we therefore assumed ethnicity as Caucasian/white for all Nivel-PCD subjects. We chose this approach in accordance with previous work and because the CHARGE-AF formula results in a prediction of an individual’s absolute 5-year AF risk. Leaving ethnicity out of the formula would lead to a systematic underestimation of absolute risk by the model.^21^

We assessed the relative contribution of each CHARGE-AF variable to an increase in baseline CHARGE-AF score by multiplying the mean value of each risk factor by its CHARGE-AF coefficient within successive strata of baseline CHARGE-AF risk.

### Statistical analysis

We reported continuous variables as means±SD, ordinal variables as median and IQR, and dichotomous variables as number and percentages. We assessed differences in baseline parameters using the unpaired t-test with Welch’s approximation, the Wilcoxon rank-sum test and the χ^2^ test where appropriate. We assessed significance in all analyses at the 0.05 level.

We estimated the cumulative 5-year AF incidence using survival analysis and presented it as number and percentages as well as incidence per 1000 person years using survival-time analysis. We plotted the cumulative AF incidence using a Kaplan-Meier failure plot.

In validation of the CHARGE-AF model for 5-year AF risk, we assessed discrimination by the C-statistic and 95% CI. We assessed calibration by the calibration plot according to deciles of baseline CHARGE-AF risk,^22^ by the calibration slope of the linear predictor and its 95% CI^22^ and by the Hosmer-Lemeshow goodness-of-fit test modified for survival analyses by D’Agostino and Nam.^23^ A Nam-D’Agostino χ^2^ with p value <0.05 indicated insufficient calibration.^24^ A calibration slope significantly smaller than 1 indicated overfitting of the CHARGE-AF model when applied to our cohort.^22^ Finally, we assessed calibration by the Kaplan-Meier failure function stratified according to baseline CHARGE-AF risk. For this, we used categories <2.5%, 2.5%–5% and >5% predicted risk in accordance with the original CHARGE-AF publication.^5^

We compared CHARGE-AF’s discriminatory abilities for risk of newly diagnosed AF with that of two other easily obtainable predictors that have previously been shown to predictive of new AF: age alone as continuous linear variable and the CHA_2_DS_2_-VASc score^25^ as a categorical variable.^4 6 26–29^ We assessed net reclassification improvement (NRI) by the NRI index and 95% CI for 5-year AF of CHARGE-AF versus age alone as well as CHARGE-AF versus CHA_2_DS_2_-VASc using 200 bootstrap samples in low, intermediate and high AF risk categories with cut-offs at 2.5% and 5% predicted AF risk.^22^ Data for age and CHA_2_DS_2_-VASc score were complete in all participants.

We performed stratified analyses according to age, sex and CHA_2_DS_2_-VASc score in all validation analyses in order to assess whether CHARGE-AF, CHA_2_DS_2_-VASc score and age would perform better among clinically relevant subgroups, and whether different predictors for newly diagnosed AF outperformed others in any of these subgroups.

Finally, we assessed the clinical implications of applying different cut-offs for dichotomisation of baseline CHARGE-AF risk into high-risk and low-risk groups. We applied cut-offs 2.5%, 5% and 10% baseline CHARGE-AF risk and assessed for each cut-off: the proportion of patients that would be counted as high risk; the proportion of total 5-year AF cases that would be among high-risk patients; 5-year AF incidence among those counted as high-risk patients; the proportion of high-risk patients with a CHA_2_DS_2_-VASc score ≥2 (corresponding with the need for oral anticoagulation therapy^2^); and the proportion of high-risk 5-year AF cases with a CHA_2_DS_2_-VASc score ≥2. In order to formally test whether the applied cut-offs were able to discriminate between high and low risk of 5-year AF incidence, we provided the unadjusted HR for 5-year AF incidence of high-risk patients with low-risk patients as reference using a Cox proportional hazards model.

We used Stata V.15.0^30^ and R V.1.1.463^31^ using the haven, nricens, polspline, rms, survival and survminer packages for our analyses.

### Ethics and study approval

Dutch law allows the use of EHRs for research purposes under certain conditions. According to this legislation, neither obtaining informed consent from patients nor approval by a medical ethics committee is obligatory for this type of observational studies containing no directly identifiable data (Dutch Civil Law, Article 7:458).^17^

## Results

We included 668 955 patients aged ≥40 years from 328 Nivel-PCD practices with follow-up data available for ≥1 year after baseline. Of these, 551 655 patients had missing data for ≥1 of the CHARGE-AF measurements height, weight, SBP and DBP during 2013. Of the 117 300 patients with complete CHARGE-AF baseline data, 5825 (4.97%) had prevalent AF at baseline. The remaining 111 475 patients free of AF and with complete CHARGE-AF variables at baseline (17.2% of all patients aged ≥40 years and free of AF) constituted the validation sample of complete cases (see study flowchart in online supplemental figure 1).

### Patients with complete CHARGE-AF baseline data

Among complete cases, mean age was 65.5±11.4 years, 52.5% were female and median CHA_2_DS_2_-VASc was 3 (IQR 2–4) (table 1). The distribution of baseline CHARGE-AF risk was skewed with more than half of all patients with complete baseline CHARGE-AF data having a predicted 5-year AF risk <5% (online supplemental figure 2, panel A). Age was the major factor driving an increase in baseline CHARGE-AF risk (online supplemental figure 2, panel B).

**Table 1:** Baseline characteristics of the study sample with complete baseline CHARGE-AF data

	All (n=111 475)	AF during follow-up (n=5264)	No AF during follow-up (n=106 211)	P value for difference*
Age, years	65.5±11.4	73.1±9.4	65.2±11.4	<0.001
Female	58 549 (52.5%)	2572 (48.9%)	55 977 (52.7%)	<0.001
SBP, mm Hg	137.3±16.3	139.5±17.3	137.2±16.2	<0.001
DBP, mm Hg	80.5±10.5	78.8±10.8	80.6±10.5	<0.001
Height, cm	170.0±9.9	170.3±9.9	170.0±9.9	0.01
Weight, kg	82.5±16.8	83.8±17.2	82.4±16.8	<0.001
Antihypertensive medication	79 057 (70.9)	4494 (85.4)	74 563 (70.2)	<0.001
Hypertension	74 149 (66.5)	3864 (73.4)	70 285 (66.2)	<0.001
Diabetes mellitus	47 557 (42.7)	2514 (47.8)	45 043 (42.4)	<0.001
Heart failure	4693 (4.2)	562 (10.7)	4131 (3.9)	<0.001
Myocardial infarction	5404 (4.9)	391 (7.4)	5013 (4.7)	<0.001
Current smoking	15 774 (14.2)	600 (11.4)	15 174 (14.3)	<0.001
Stroke	7462 (6.7)	472 (9.0)	6990 (6.6)	<0.001
TIA	3339 (3.0)	224 (4.3)	3115 (2.9)	<0.001
Pulmonary embolism	506 (0.5)	31 (0.6)	475 (0.4)	0.14
Angina pectoris	10 167 (9.1)	750 (14.3)	9417 (8.9)	<0.001
CHA_2_DS_2_-VASc	3 (IQR 2–4)	4 (IQR 3–5)	3 (IQR 2–4)	<0.001
CHA_2_DS_2_-VASc≥2	88 538 (79.4)	4866 (92.4)	83 672 (78.8)	<0.001
Asthma	13 262 (11.9)	652 (12.4)	12 610 (11.9)	0.26
COPD	12 523 (11.2)	879 (16.7)	11 644 (11.0)	<0.001
Atherosclerosis	6367 (5.7)	416 (7.9)	5951 (5.6)	<0.001
Hypercholesterolaemia	19 427 (17.4)	694 (13.2)	18 733 (17.6)	<0.001
Gout	7639 (6.9)	589 (11.2)	7050 (6.6)	<0.001
Enrolled in care programme				
Asthma	1846 (1.7)	77 (1.5)	1769 (1.7)	0.26
COPD	4777 (4.3)	335 (6.4)	4442 (4.2)	<0.001
Diabetes mellitus	35 640 (32.0)	1943 (36.9)	33 697 (31.7)	<0.001
Any care programme	40 468 (36.3)	2212 (42.0)	38 256 (36.0)	<0.001

Data are number (percentage), mean±SD or median (IQR).

*Difference between those with and without AF during follow-up.

AF, atrial fibrillation; CHA_2_DS_2_-VASc, congestive heart failure, hypertension, age, diabetes and previous stroke or transient ischaemic attack, vascular disease and female sex category; CHARGE-AF, Cohorts for Heart and Aging Research in Genomic Epidemiology-atrial fibrillation; COPD, chronic obstructive pulmonary disorder; DBP, diastolic blood pressure; IQR, interquartile range; SBP, systolic blood pressure; TIA, transient ischaemic attack.

Compared with those who remained free of AF, patients who were diagnosed with new AF during follow-up were older and had higher overall cardiovascular burden, except for DBP, burden of hypercholesterolaemia and proportion of current smokers that were lower. For a comparison between patients with and those without complete baseline CHARGE-AF data, see online supplemental results.

### AF incidence and follow-up

There were 5264 cases of new AF among complete CHARGE-AF cases during the 5-year follow-up window (4.7%; 13.6/1000 person-years; see online supplemental figure 3, panel A, for the Kaplan-Meier plot). Mean follow-up in the sample was 3.5±1.7 years. Main reason for loss to follow-up was practices’ data being excluded from further analysis due to low quality data (see online supplemental figure 3, panel B, for the number of practices and patients at risk during follow-up).

### CHARGE-AF validation

**Figure 1:**
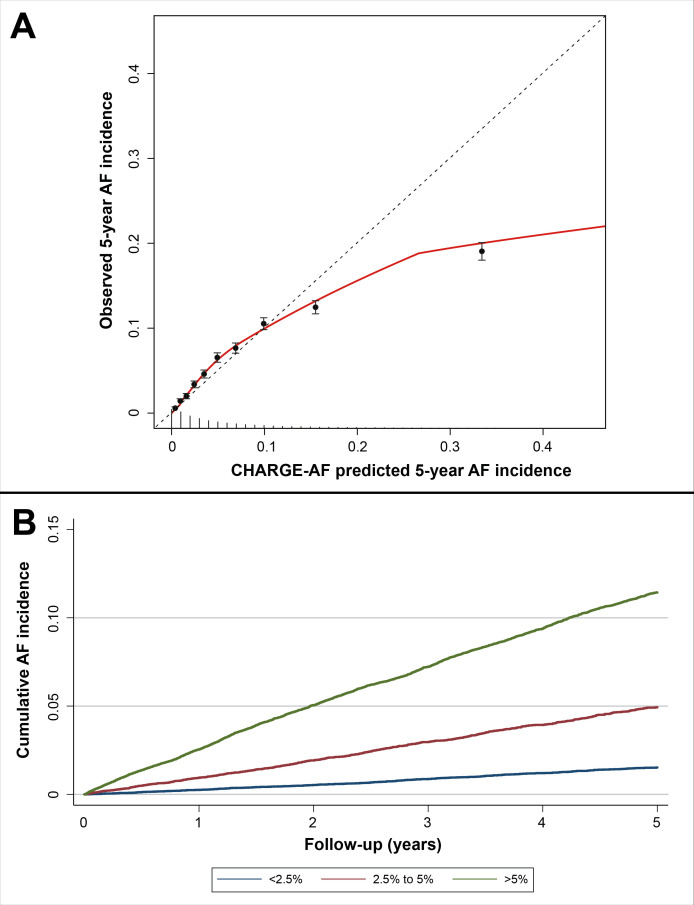
Panel A: calibration plot for CHARGE-AF. The points indicate intersects of observed and expected for each decile of baseline CHARGE-AF risk, with brackets indicating the 95% CI of observed AF probability during 5-year follow-up in each decile. The red line indicates the trend for CHARGE-AF calibration in the sample. When the intersect of observed and expected AF incidence exceeds the dotted line, this indicates underestimation of AF risk by CHARGE-AF for that decile. When the intersect of observed and expected AF incidence is below the dotted line, this indicates overestimation of AF risk by CHARGE-AF for that decile. The spikes on the x-axis indicate the distribution of AF-free survivors by CHARGE-AF risk; panel B: Kaplan-Meier plot of AF incidence stratified according to baseline CHARGE-AF predicted risk categories <2.5%, 2.5%–5% and >5%. AF, atrial fibrillation; CHARGE-AF, Cohorts for Heart and Aging Research in Genomic Epidemiology-atrial fibrillation.

Validation of CHARGE-AF among all patients with complete baseline CHARGE-AF data resulted in a C-statistic of 0.736 (95% CI 0.727 to 0.744), a Nam-D’Agostino χ^2^ of 901.8 (p<0.001) and a calibration slope of 0.69 (95% CI 0.67 to 0.71) (table 2). The calibration plot showed a slight underestimation of AF risk among lower deciles of CHARGE-risk but strong overestimation of AF risk in the higher CHARGE-AF deciles (figure 1, panel A). The Kaplan-Meier plot stratified by risk categories <2.5%, 2.5%–5% and >5% CHARGE-AF predicted 5-year risk indicated an accurate estimation of observed 5-year AF risk in the overall sample of complete cases (figure 1, panel B).

**Table 2:** Validation of CHARGE-AF, CHA_2_DS_2_-VASc and age alone as predictors for 5-year AF incidence among patients with complete baseline CHARGE-AF data (n=111 475)

	CHARGE-AF	CHA_2_DS_2_-VASc	Age alone
**All (n=111 475; 5264 AF cases**)			
C-statistic (95% CI)	0.736 (0.727 to 0.744)	0.669 (0.661 to 0.677)	0.716 (0.708 to 0.724)
Nam-D’Agostino χ^2^ (p value)	901.8 (p<0.001)	–	–
Calibration slope (95% CI)	0.69 (0.67 to 0.71)	–	–
**Age ≥65 years (n=60 528; 4356 AF cases)**			
C-statistic (95% CI)	0.646 (0.637 to 0.655)	0.581 (0.572 to 0.590)	0.615 (0.606 to 0.624)
Nam-D’Agostino χ^2^ (p value)	907.2 (p<0.001)	–	–
Calibration slope (95% CI)	0.58 (0.54 to 0.61)	–	–
**Age <65 years (n=50 947; 908 AF cases**)			
C-statistic (95% CI)	0.706 (0.686 to 0.725)	0.543 (0.525 to 0.561)	0.652 (0.633 to 0.671)
Nam-D’Agostino χ^2^ (p value)	38.8 (p<0.001)	–	–
Calibration slope (95% CI)	0.93 (0.84 to 1.01)	–	–
**Men (n=52 926; 2692 AF cases**)			
C-statistic (95% CI)	0.718 (0.707 to 0.729)	0.672 (0.661 to 0.683)	0.702 (0.691 to 0.713)
Nam-D’Agostino χ^2^ (p value)	542.7 (p<0.001)	–	–
Calibration slope (95% CI)	0.65 (0.62 to 0.69)	–	–
**Women (n=58 549; 2572 AF cases**)			
C-statistic (95% CI)	0.751 (0.740 to 0.763)	0.706 (0.695 to 0.717)	0.736 (0.724 to 0.750)
Nam-D’Agostino χ^2^ (p value)	381.6 (p<0.001)	–	–
Calibration slope (95% CI)	0.72 (0.69 to 0.76)	–	–
**CHA_2_DS_2_-VASc≥2 (n=88 538; 4866 AF cases**)			
C-statistic (95% CI)	0.711 (0.702 to 0.719)	0.636 (0.628 to 0.644)	0.688 (0.680 to 0.697)
Nam-D’Agostino χ^2^ (p value)	866.0 (p<0.001)	–	–
Calibration slope (95% CI)	0.66 (0.63 to 0.68)	–	–
**CHA_2_DS_2_-VASc<2 (n=22 937; 398 AF cases**)			
C-statistic (95% CI)	0.723 (0.694 to 0.752)	0.521 (0.501 to 0.541)	0.680 (0.649 to 0.707)
Nam-D’Agostino χ^2^ (p value)	20.4 (p=0.02)	–	–
Calibration slope (95% CI)	0.99 (0.86 to 1.12)	–	–

CHA_2_DS_2_-VASc, congestive heart failure, hypertension, age, diabetes and previous stroke or transient ischaemic attack, vascular disease and female sex category; CHARGE-AF, Cohorts for Heart and Aging Research in Genomic Epidemiology model for atrial fibrillation.

CHARGE-AF showed superior discrimination to CHA_2_DS_2_-VASc as well as age alone as the predictor in both the overall and all stratified analyses. Results of the stratified analyses on CHARGE-AF are shown in the online supplementary results. CHARGE-AF resulted in significant reclassification improvement versus both CHA_2_DS_2_-VASc (NRI index: 0.24; 95% CI 0.22 to 0.25) and age alone (NRI index: 0.05; 95% CI 0.04 to 0.06).

### Application of different CHARGE-AF cut-offs

**Figure 2:**
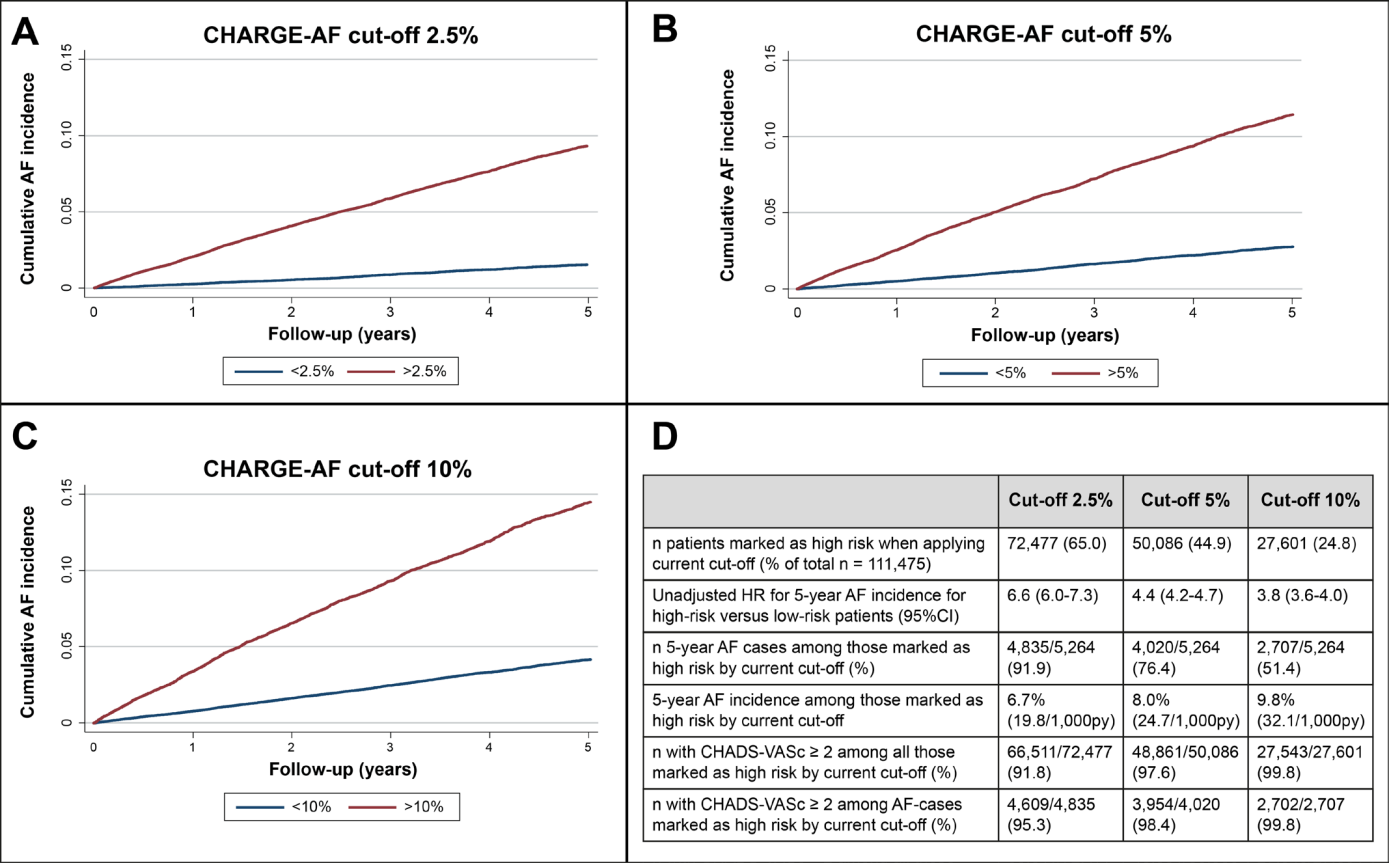
Panel A: Kaplan-Meier (KM) plot of AF incidence dichotomised according to baseline CHARGE-AF predicted risk cut-off 2.5%; panel B: KM plot of AF incidence dichotomised according to baseline CHARGE-AF predicted risk cut-off 5%; panel C: KM plot of AF incidence dichotomised according to baseline CHARGE-AF predicted risk cut-off 10%; panel D: table of outcomes if CHARGE-AF risk cut-offs 2.5%, 5% and 10%, respectively, had been applied for patient selection. AF, atrial fibrillation; CHA_2_DS_2_-VASc, congestive heart failure, hypertension, age, diabetes and previous stroke or transient ischaemic attack, vascular disease and female sex category; CHARGE-AF, cohorts for Heart and Ageing Research in Genomic Epidemiology model for atrial fibrillation; PY, person years; Nivel-PCD, Netherlands Institute for Health Services Research Primary Care Database.

Figure 2 shows the analysis on dichotomisation of CHARGE-AF risk at cut-offs 2.5%, 5% and 10%. The high-risk groups showed significantly higher AF incidence over time in all comparisons as assessed by the unadjusted HRs for high-risk versus low-risk patients. Cut-offs at 2.5%, 5% and 10% CHARGE-AF risk would have classified 65%, 45% and 25% of patients with complete CHARGE-AF baseline data as ‘high risk’, respectively. Routine care 5-year AF incidence among the high-risk patients at these cut-offs was 6.7%, 8.0% and 9.8%, respectively. In all high-risk groups, >95% observed AF cases had CHA_2_DS_2_-VASc ≥2 at baseline (p<0.001 for difference with proportion of CHA_2_DS_2_-VASc ≥2 among low-risk AF cases in all comparisons).

## Discussion

In a routine primary care EHR database representative of the Netherlands, one in six patients aged 40 years and older was free of AF and had complete baseline CHARGE-AF data. These patients had significantly higher 5-year AF incidence and cardiovascular morbidity than those with ≥1 missing CHARGE-AF variables. Validation of CHARGE-AF among complete cases showed that despite overestimation of absolute 5-year AF risk in those with the highest baseline CHARGE-AF scores, the model had overall sufficient discrimination for 5-year AF risk and was able to accurately group patients according to predefined risk categories. CHARGE-AF had superior discrimination for 5-year risk of AF compared with CHA_2_DS_2_-VASc and age alone. Explorative analyses on the application of different CHARGE-AF cut-offs for patient selection indicated that cut-offs at 2.5%, 5% and 10% all have potential merits for use in AF risk stratification.

### Clinical implications

Outcomes of this work are relevant to the prospect of using clinical risk models as triage test for AF screening, while maintaining low cost in their risk assessment efforts. We showed that those with complete CHARGE-AF variables as per routine primary care constitute a small but highly relevant subset for AF screening. The model’s high accuracy in predicting absolute 5-year risk for predefined risk categories suggests that the model can be used to reliably differentiate between low and high AF risk among complete cases. Moreover, CHARGE-AF outperformed two other predictors that have been employed to select for AF screening eligibility, as assessed by both the C-statistic and NRI index. This work therefore encourages researchers in the field of community AF screening to consider CHARGE-AF as a triage test for patient selection.

We provided data on how the choice for a baseline CHARGE-AF cut-off for classifying patients as ‘high risk’ could translate into actual patient selection for screening. The sensitivity of ‘baseline CHARGE-AF’ as a triage test for 5-year observed new AF ranged between 51% at CHARGE-AF cut-off 0.1% and 92% at CHARGE-AF cut-off 0.025. Since these findings are based on simple routine care EHR data acquired without imputation or text mining techniques, CHARGE-AF showed its potential for low-cost automated, remote AF risk stratification. This suggests a lower need for a baseline visit prior to screening. The model could also be used as an alert for clinicians to check for AF in the subset of patients with complete data through routine care.

We emphasise that the outcome in our work was 5-year risk of an AF diagnosis acquired through routine care. To our knowledge, there have been no clinical studies on the efficacy of CHARGE-AF as a triage test for patient selection for screening. Although our work does not provide concrete recommendations to practising GPs on whether and how to best use CHARGE-AF in selecting patients for further rhythm analysis, it points to CHARGE-AF as a model with the highest potential for this purpose.

### Comparison with previous work

This study diverges from previous CHARGE-AF validation studies in that it made an explicit attempt to bridge the gap between model validation and subsequent application as a tool for patient selection in community AF screening. To our knowledge, we were the first to provide detailed information on how selecting at different cut-offs would translate into numbers of patients to be screened and percentage of AF yield to be expected in a large routine primary care dataset.

The C-statistic for CHARGE-AF in our study (0.74) was lower than in the aggregate CHARGE-AF derivation cohorts (0.77) but higher than the summary C-statistic in a recent meta-analysis of CHARGE-AF for 5-year AF risk in community cohorts (0.72).^5 11^ Possible explanations for difference with the original CHARGE-AF article are that the model was calibrated to fit the derivation data, that our dataset had a lower percentage of women in whom CHARGE-AF performed better than in men and that the ethnic diversity was lower in Nivel-PCD. Applying the same age restrictions to our dataset as were used in the derivation article (46–94 years) resulted in the same C-statistic as the current overall analysis (data not shown).

A recent study validated CHARGE and CHA_2_DS_2_-VASc based on a large routine care EHR dataset from seven hospitals in the USA from which they excluded patients with non-complete measurement data.^18^ Results of validation of CHARGE-AF and CHA_2_DS_2_-VASc were similar to ours. The main difference between this study and ours is the population. Since Dutch primary care EHR data covers all non-institutionalised inhabitants, with all secondary care facilities reporting back to GPs, Nivel-PCD is likely to have a wider coverage of the population than a regional agglomeration of hospitals. The percentage of patients with complete measurements, however, was greater in Hulme *et al*’s^18^ hospital-derived dataset where measurements may be more routinely taken. Both studies, however, provide evidence that routine care data can be used to assess risk of AF in patients with complete measurement data at baseline, with each study having its own merits in terms of generalisability to different care settings.

Although our patient selection differed from the derivation study as well as previous validation studies that were performed in largely unselected community cohorts, a number of observations are common among validation studies of CHARGE-AF, age alone and CHA_2_DS_2_-VASc for new AF. Mainly, these studies, like ours, found that CHARGE-AF outperformed CHA_2_DS_2_-VASc and age alone as predictors for new AF and that CHARGE-AF showed higher C-statistics among lower risk subgroups within their sample.^4–10 26–29 32–34^

Our study corroborates the findings that patients with complete recent baseline measurement data as per routine care were older and had higher burden of cardiovascular comorbidity than those with missing measurements.^12^ Our study expands on that by showing that having complete measurements through routine primary care is also associated with higher 5-year risk of AF.

We were unable to validate a number of other models developed for AF risk prediction in community cohorts due to restrictions in data availability in Dutch primary care EHRs.^6 8 18 26 35 36^ We refrained from recalibration and augmentation of CHARGE-AF to better fit our sample, since our aim was to validate CHARGE-AF, not to improve its risk prediction in a specific population.^4 5 7 10 27 32–34 37^

### Future work

Our work relied heavily on the assumption that AF risk through routine care is correlated with AF yield through active screening. Although there are few studies to assess the validity of this hypothesis, one recent pilot study that selected individuals with both age ≥65 years and high CHA_2_DS_2_-VASc score for screening with continuous ECG monitoring found promising results.^38^ Post hoc analyses on the added value of multivariable risk models in previous AF screening studies would be welcomed.

Our work shows that higher completeness of primary care EHR data is needed. Since such data completeness will likely not be achieved in the foreseeable future, research should focus on ways of handling missing data in primary care EHRs while still achieving accurate risk prediction. Until then, models that do not rely on measurement variables may be the model of choice for remote, automatic AF risk assessment in primary care settings. Finally, the ethical implications of using EHR data to remotely brand individuals as ‘at high risk of AF and stroke’ deserve further research.^3^

### Strengths and limitations

This work had a number of strengths. First, our validation of CHARGE-AF in patients with complete data through routine primary care enabled an assessment of CHARGE-AF’s merits as a potential triage test for AF screening without the need for a resource-intensive baseline visit for data collection. Second, given the use of a large dataset that encompasses a representative sample of primary care patients in the Netherlands, and considering the role of GPs in the Netherlands where all inhabitants are registered at a GP and where all secondary care providers report health outcomes back to GPs, results from this study are likely generalisable to similar settings.^20^ Third, we included a comparison of patients with and without complete baseline CHARGE-AF measurements. This enabled us to show that patients with complete baseline parameters had higher AF risk and higher cardiovascular comorbidity and more often had a CHA_2_DS_2_-VASc score ≥2. An AF diagnosis in these patients is therefore both more likely and more often relevant in terms of anticoagulation initiation.^2^ Finally, we provided researchers interested in using CHARGE-AF as a selection tool for AF screening among complete cases with ample data to assess which baseline CHARGE-AF cut-off may be most viable for such purposes.

Our study’s primary strength was also its most prominent limitation. Due to its restriction to patients with complete CHARGE-AF measurements, results of this study are not generalisable to the community at large. Additional work is therefore required to assess how CHARGE-AF can be used to reliably assess risk for incident AF in the larger community while still refraining from the need to perform baseline visits. Second, the nature of a routine primary care database dictates that diagnosis and correct registration of morbidities had been at treating physicians’ discretion. Most notably, this may increase the risk of verification bias in diagnosing incident AF as well as underestimation of prevalence of baseline comorbidities.^39 40^ Third, one of CHARGE-AF’s variables—ethnicity—was missing altogether from the database due to restrictions in Dutch primary healthcare regulations. Although our evaluation of the relative contribution of variables to increments in baseline risk showed ethnicity to play only a minor role in overall AF risk assessment when assumed as Caucasian/white in all individuals, it is unclear how information on this variable might have influenced the validity of predictions in non-Caucasian individuals. Finally, it is unclear whether the classification of AF and MI diagnoses as non-chronic episodes in Nivel-PCD, with a patient’s AF or MI episode being inactivated after a contact-free period of 1 year, may have affected AF prevalence and CHARGE-AF score before baseline and AF incidence during follow-up.^16^ Prior work on Nivel-PCD showed that extending this period from 1 to 2 years did not lead to significantly different incidence rates.^16^ We sought to further ameliorate this limitations by using a 1-year baseline window, which has been shown to lead to a more accurate representation of disease prevalence in routine care EHRs than point prevalence.^20^ We hereby effectively extended the non-contact window after which AF and MI patients would become false-negative from 1 to 2 years before baseline.

## Data Availability

Data are deidentified routine primary care electronic health records licensed by the Netherlands Institute for Health Services Research Primary Care Database. For requests for and information on data usage: directie@nivel.nl.

## References

[1] Magnussen C, Niiranen TJ, Ojeda FM, et al. Sex differences and similarities in atrial fibrillation epidemiology, risk factors, and mortality in community cohorts: results from the BiomarCaRE Consortium (biomarker for cardiovascular risk assessment in Europe). Circulation 2017;136:1588–97. 10.1161/CIRCULATIONAHA.117.028981 29038167 PMC5657474

[2] Kirchhof P, Benussi S, Kotecha D, et al. 2016 ESC guidelines for the management of atrial fibrillation developed in collaboration with EACTS. Europace 2016;18:1609–78. 10.1093/europace/euw295 27567465

[3] Freedman B, Camm J, Calkins H, et al. Screening for atrial fibrillation: a report of the AF-SCREEN international collaboration. Circulation 2017;135:1851. 10.1161/CIRCULATIONAHA.116.026693 28483832

[4] Linker DT, Murphy TB, Mokdad AH. Selective screening for atrial fibrillation using multivariable risk models. Heart 2018;104:1492–9. 10.1136/heartjnl-2017-312686 29593077

[5] Alonso A, Krijthe BP, Aspelund T, et al. Simple risk model predicts incidence of atrial fibrillation in a racially and geographically diverse population: the CHARGE-AF consortium. J Am Heart Assoc 2013;2:e000102. 10.1161/JAHA.112.000102 23537808 PMC3647274

[6] Li Y-G, Pastori D, Farcomeni A, et al. A Simple Clinical Risk Score (C_2_HEST) for Predicting Incident Atrial Fibrillation in Asian Subjects: Derivation in 471,446 Chinese Subjects, With Internal Validation and External Application in 451,199 Korean Subjects. Chest 2019;155:510–8. 10.1016/j.chest.2018.09.011 30292759 PMC6437029

[7] Berntsson J, Smith JG, Nilsson PM, et al. Pro-Atrial natriuretic peptide and prediction of atrial fibrillation and stroke: the Malmö Preventive Project. Eur J Prev Cardiol 2017;24:788–95. 10.1177/2047487317693948 28195503

[8] Kokubo Y, Watanabe M, Higashiyama A, et al. Development of a Basic Risk Score for Incident Atrial Fibrillation in a Japanese General Population - The Suita Study. Circ J 2017;81:1580–8. 10.1253/circj.CJ-17-0277 28539563

[9] Pfister R, Brägelmann J, Michels G, et al. Performance of the CHARGE-AF risk model for incident atrial fibrillation in the EPIC Norfolk cohort. Eur J Prev Cardiol 2015;22:932–9. 10.1177/2047487314544045 25059930

[10] Svennberg E, Lindahl B, Berglund L, et al. NT-proBNP is a powerful predictor for incident atrial fibrillation - Validation of a multimarker approach. Int J Cardiol 2016;223:74–81. 10.1016/j.ijcard.2016.08.001 27541645

[11] Himmelreich JCL, Veelers L, Lucassen WAM, et al. Prediction models for atrial fibrillation applicable in the community: a systematic review and meta-analysis. Europace 2020;22:684–94. 10.1093/europace/euaa005 32011689 PMC7526764

[12] Marston L, Carpenter JR, Walters KR, et al. Issues in multiple imputation of missing data for large general practice clinical databases. Pharmacoepidemiol Drug Saf 2010;19:618–26. 10.1002/pds.1934 20306452

[13] Verberne LDM, Nielen MMJ, Leemrijse CJ, et al. Recording of weight in electronic health records: an observational study in general practice. BMC Fam Pract 2018;19:174. 10.1186/s12875-018-0863-x 30447691 PMC6240309

[14] Collins GS, Reitsma JB, Altman DG, et al. Transparent reporting of a multivariable prediction model for individual prognosis or diagnosis (TRIPOD): the TRIPOD statement. Ann Intern Med 2015;162:55–63. 10.7326/M14-0697 25560714

[15] Lamberts H, Wood M. International classification of primary care. 1st ed. Oxford: Oxford University Press, 1987.

[16] Nielen MMJ, Spronk I, Davids R, et al. Estimating morbidity rates based on routine electronic health records in primary care: observational study. JMIR Med Inform 2019;7:e11929. 10.2196/11929 31350839 PMC6688441

[17] Nivel. Netherlands Institute for health services research, 2018. Available: https://www.nivel.nl/en

[18] Hulme OL, Khurshid S, Weng L-C, et al. Development and validation of a prediction model for atrial fibrillation using electronic health records. JACC Clin Electrophysiol 2019;5:1331–41. 10.1016/j.jacep.2019.07.016 31753441 PMC6884135

[19] Vermond RA, Geelhoed B, Verweij N, et al. Incidence of atrial fibrillation and relationship with cardiovascular events, heart failure, and mortality: a community-based study from the Netherlands. J Am Coll Cardiol 2015;66:1000–7. 10.1016/j.jacc.2015.06.1314 26314526

[20] Spronk I, Korevaar JC, Poos R, et al. Calculating incidence rates and prevalence proportions: not as simple as it seems. BMC Public Health 2019;19:512. 10.1186/s12889-019-6820-3 31060532 PMC6501456

[21] Held U, Kessels A, Garcia Aymerich J, et al. Methods for handling missing variables in risk prediction models. Am J Epidemiol 2016;184:545–51. 10.1093/aje/kwv346 27630143

[22] Steyerberg EW, Vickers AJ, Cook NR, et al. Assessing the performance of prediction models: a framework for traditional and novel measures. Epidemiology 2010;21:128–38. 10.1097/EDE.0b013e3181c30fb2 20010215 PMC3575184

[23] D’Agostino R, Nam BH. Evaluation of the performance of survival analysis models: Discrimination and calibration measures. In: Balakrishnan N, Rao CR, eds. Handbook of statistics. Amsterdam: Elsevier, 2004: 1–25.

[24] Demler OV, Paynter NP, Cook NR. Tests of calibration and goodness-of-fit in the survival setting. Stat Med 2015;34:1659–80. 10.1002/sim.6428 25684707 PMC4555993

[25] Lip GYH, Nieuwlaat R, Pisters R, et al. Refining clinical risk stratification for predicting stroke and thromboembolism in atrial fibrillation using a novel risk factor-based approach: the Euro heart survey on atrial fibrillation. Chest 2010;137:263–72. 10.1378/chest.09-1584 19762550

[26] Everett BM, Cook NR, Conen D, et al. Novel genetic markers improve measures of atrial fibrillation risk prediction. Eur Heart J 2013;34:2243–51. 10.1093/eurheartj/eht033 23444395 PMC3730133

[27] Alonso A, Roetker NS, Soliman EZ, et al. Prediction of atrial fibrillation in a racially diverse cohort: the Multi‐Ethnic Study of Atherosclerosis (MESA). J Am Heart Assoc 2016;5:1–8. 10.1161/JAHA.115.003077 PMC4802458 26908413

[28] Christophersen IE, Yin X, Larson MG, et al. A comparison of the CHARGE-AF and the CHA2DS2-VASc risk scores for prediction of atrial fibrillation in the Framingham Heart Study. Am Heart J 2016;178:45–54. 10.1016/j.ahj.2016.05.004 27502851 PMC5344697

[29] Saliba W, Gronich N, Barnett-Griness O, et al. Usefulness of CHADS2 and CHA2DS2-VASc scores in the prediction of new-onset atrial fibrillation: a population-based study. Am J Med 2016;129:843–9. 10.1016/j.amjmed.2016.02.029 27012854

[30] StataCorp. Stata statistical software: release 15. College Station, TX: StataCorp LLC, 2017.

[31] R Core Team. R: a language and environment for statistical computing. R foundation for statistical computing, Vienna, Austria. URL, 2019. Available: https://www.R-project.org/

[32] Chaker L, Heeringa J, Dehghan A, et al. Normal thyroid function and the risk of atrial fibrillation: the Rotterdam study. J Clin Endocrinol Metab 2015;100:3718–24. 10.1210/jc.2015-2480 26262438

[33] Maheshwari A, Norby FL, Soliman EZ, et al. Refining prediction of atrial fibrillation risk in the general population with analysis of P-Wave axis (from the Atherosclerosis Risk in Communities Study). Am J Cardiol 2017;120:1980–4. 10.1016/j.amjcard.2017.08.015 28941601 PMC9835766

[34] Sinner MF, Stepas KA, Moser CB, et al. B-type natriuretic peptide and C-reactive protein in the prediction of atrial fibrillation risk: the CHARGE-AF Consortium of community-based cohort studies. Europace 2014;16:1426–33. 10.1093/europace/euu175 25037055 PMC4197895

[35] Schnabel RB, Sullivan LM, Levy D, et al. Development of a risk score for atrial fibrillation (Framingham heart study): a community-based cohort study. Lancet 2009;373:739–45. 10.1016/S0140-6736(09)60443-8 19249635 PMC2764235

[36] Hamada R, Muto S. Simple risk model and score for predicting of incident atrial fibrillation in Japanese. J Cardiol 2019;73:65–72. 10.1016/j.jjcc.2018.06.005 30064946

[37] Schnabel RB, Aspelund T, Li G, et al. Validation of an atrial fibrillation risk algorithm in whites and African Americans. Arch Intern Med 2010;170:1909–17. 10.1001/archinternmed.2010.434 21098350 PMC3021784

[38] Pessoa-Amorim G, Casadei B, Jones NR, et al. Active monitoring for atrial fibrillation (AMALFI): protocol and pilot from a mail-based randomized trial of screening for subclinical atrial fibrillation in high-risk individuals. ESC Heart & Stroke 2020;9.

[39] Barkhuysen P, de Grauw W, Akkermans R, et al. Is the quality of data in an electronic medical record sufficient for assessing the quality of primary care? J Am Med Inform Assoc 2014;21:692–8. 10.1136/amiajnl-2012-001479 24145818 PMC4078269

[40] de Groot JAH, Bossuyt PMM, Reitsma JB, et al. Verification problems in diagnostic accuracy studies: consequences and solutions. BMJ 2011;343:d4770. 10.1136/bmj.d4770 21810869

