# Correction: *CHARGE-AF in a national routineprimary care electronic health recordsdatabase in the Netherlands: validationfor 5-year risk of atrial fibrillation andimplications for patient selection inatrial fibrillation screening*

**DOI:** 10.1136/openhrt-2020-001459corr1

**Published:** 2021-10-18

Himmelreich JCL, Lucassen WAM, Harskamp RE, *et al*. CHARGE-AF in a national routine primary care electronic health records database in the Netherlands: validation for 5-year risk of atrial fibrillation and implications for patient selection in atrial fibrillation screening. *Open Heart* 2021;8:e001459. doi: 10.1136/openhrt-2020-001459

This article has been corrected since it was first published. The provenance and peer review statement has been included.

