# Supplementary material for: CHARGE-AF in a national routine primary care electronic health records database in the Netherlands: validation for 5-year risk of atrial fibrillation and implications for patient selection in atrial fibrillation screening
Source: Open Heart. 2021 Jan 18;8(1):e001459. doi: 10.1136/openhrt-2020-001459 (PMC7816907; doi:10.1136/openhrt-2020-001459)
Supplement: Supplementary data [file openhrt-2020-001459supp001.pdf]

Supplement to Himmelreich et al. "CHARGE-AF in complete cases from a national routine primary care health records database in the Netherlands: validation for 5-year risk of atrial fibrillation and implications for patient selection in atrial fibrillation screening"

Supplementary Methods

Nivel-PCD episodes of illness construction in current dataset

Episodes of illness that are deemed 'chronic' in Nivel-PCD, including e.g. hypertension and diabetes mellitus (DM), remain active throughout extractions. This allowed for inclusion of all recorded chronic episodes of illness prior to 1 January, 2014, including those with their latest GP encounters prior to calendar year 2013. Episodes of illness that are classified as 'long-lasting reversible diseases' in Nivel-PCD, a category that includes AF and MI, are available in an annual extraction if the last GP encounter was up to 1 year prior to extraction. We were thus able to include all long-lasting reversible diseases of which a patient's EHR contained a recorded GP encounter (physical or administrative) on or later than 1 January, 2012. Prior Nivel-PCD analyses have shown that extension of this 1-year contact-free interval does not lead to significant differences in long-lasting reversible disease incidence.(1)

Operational variable definitions

- Atrial fibrillation (AF): entry of ICPC-1 code K78 (AF/flutter) and/or data codes 3451 (treating physician for AF) or 3838 (enrolment in care program for AF);
- Age: the discrete number of years attained in the year 2013 since year of birth;
- Sex: male or female;
- Systolic blood pressure (SBP): latest recording in 2013 of data codes 1744 (SBP), 2055 (SBP home measurement), 2668 (mean SBP in 24-hour measurement), 3336 (mean SBP in 30-minute measurement), 1745 (SBP lying down), 2189 (SBP standing), or 1794 (SBP of the arm when used for ankle-brachial index test). We applied a hierarchy in which code to use, in the order of aforementioned data codes. We first looked at entries for data code 1744 and when available we used the latest entry in 2013. If there was no entry for data code 1744, we looked at entries for data code 2055. If there was no entry for data code 2055, we looked at data code 2668, etc. until data code 1794. In order to prevent inclusion of values erroneously entered by GP personnel, we included only SBP values 25-250mmHg;
- Diastolic blood pressure (DBP): latest recording in 2013 of data codes 1740 (DBP), 2056 (DBP home measurement), 2669 (mean DBP in 24-hour measurement), 3337 (mean DBP in 30-minute measurement), 1741 (DBP lying down), or 2188 (DBP standing). We applied a hierarchy in which code to use, in the order of aforementioned data codes. We first looked at entries for data code 1740 and when available we used the latest entry in 2013. If there was no entry for data code 1740, we looked at entries for data code 2056. If there was no entry for data code 2056, we looked at data code 2669, etc. until data code 2188. In order to prevent inclusion of values erroneously entered by GP personnel, we included only SBP values 25-250mmHg;
- Weight: latest recording in 2013 of data codes 357 (weight) or 2408 (weight home measurement). When entries for these data codes were absent in 2013, but data codes 560 (height) and 1272 (body mass index, BMI) were present, we calculated weight as BMI*weight^2 and used the latest recordings in 2013. In order to prevent inclusion of values erroneously entered by GP personnel, we included only weight values 30-300kg;

- Height: latest recording in 2013 of data code 560 (height). When an entry for data code 560 was absent, but data codes for weight and BMI were both present in 2013, we calculated height in centimeters as 100*√(weight/BMI) and used the latest recordings in 2013. In order to include only realistic values, and to prevent inclusion of values erroneously entered by GP personnel, we included only height values 130-230cm. Values below 130 were multiplied by 100 in order to include data entered as meters instead of centimeters. We subsequently applied the same limits of 130-230cm;
- Antihypertensive medication: ATC subcodes for C02 (antihypertensives) and/or C03 (diuretics), C04 (peripheral vasodilators), C05 (vasoprotectives), C07 (beta blocking agents), C08 (calcium channel blockers), or C9 (agents acting on the renin-angiotensin system);
- Hypertension: entry of ICPC-1 codes K86 (uncomplicated hypertension) and/or K87 (hypertension with involvement target organs) or data code 1694 (hypertension comorbidity);
- Diabetes mellitus (DM): entry of ICPC-1 code T90 (DM) and/or data code 2206 (treating physician for DM);
- Heart failure (HF): entry of ICPC-1 code K77 (HF) and/or data codes 3016 (treating physician for HF), 2722 (NYHA severity of HF symptoms) or 1643 (HF comorbidity);
- Myocardial infarction (MI): entry of ICPC-1 code K75 (acute MI) and/or data code 1693 (MI comorbidity);
- Current smoking: classified as current smoker when indicated as smoker as per data codes 1739 (smoking) and/or 1992 (number of (rolling tobacco) cigarettes per day), 1993 (number of cigarettes per day), 1996 (wants to quit smoking in short term) or 2405 (motivation to quit smoking), and not followed in time (but before 01-01-2014) by an indication of having quit smoking as per data codes 1739 (smoking) and/or 2003 (quit smoking since);
- Stroke: entry of ICPC-1 code K90 (stroke/cerebrovascular accident) and/or lab code 2132 (cerebral ischaemia history comorbidity);
- Transient ischemic attack (TIA): entry of ICPC-1 code K89 (transient cerebral ischaemia);
- Pulmonary embolism (PE): entry of ICPC-1 code K93 (PE);
- Angina pectoris: entry of ICPC-1 code K74 (angina pectoris);
- Vascular disease: entry of ICPC-1 codes K74 (angina pectoris) and/or K91 (atherosclerosis), K92 (other arterial obstruction/peripheral vascular disease) or MI as defined above;
- Congestive heart failure, Hypertension, Age, Diabetes and previous Stroke or Transient Ischaemic Attack, Vascular disease and female Sex category (CHA_2DS_2-VASc): 1 point for each of female sex, HF, hypertension, DM, vascular disease or age 65-74 years, plus 2 points for each of (stroke, TIA or PE) or age ≥75 years;
- Asthma: entry of ICPC-1 code R96 (asthma) and/or indication for asthma as per data codes 1598 (asthma diagnosed by) and/or 1599 (asthma goals attained), 1618 (medication adherence asthma), 1621 (avoids provoking factors asthma), 1716 (reason for failure to achieve asthma goals), 1776 (asthma management), 1806 (change asthma medication), 1822 (asthma severity), 1824 (asthma self-management), 1826 (appointment for asthma self-management), 1877 (asthma comorbidity), 2406 (treating physician for asthma), 3018 (adverse effects asthma medication), 3608 (degree of control in asthma management), 3338 (ACQ question 1), 3339 (ACQ question 2), 3340 (ACQ question 3), 3341 (ACQ question 4), 3345 (C-ACT question 1), 3346 (C-ACT question 2), 3347 (C-ACT question 3), 3348 (C-ACT question 4), 3349 (C-ACT question 5), 3828 (enrolment in care program for asthma);
- Chronic obstructive pulmonary disease (COPD): entry of ICPC-1 code R95 (COPD) and/or indication for COPD as per data codes 1779 (medication adherence COPD) and/or 1785 (COPD management), 1786 (causes for COPD exacerbation), 1807 (change COPD medication), 1818

(reason not to enrol in COPD care program), 1909 (reasons for not attaining COPD goals), 1911 (COPD diagnosed by), 2209 (GOLD classification COPD), 2399 (mean symptom score CCQ COPD), 2400 (mean function score CCQ COPD), 2401 (mean psychological score CCQ COPD), 2402 (mean limitations score CCQ COPD), 2407 (treating physician COPD), 2676 (cachexia COPD), 3013 (COPD disease burden), 3019 (adverse effects COPD medication);

- Atherosclerosis: entry of ICPC-1 code K91 (atherosclerosis);
- Hypercholesterolaemia: entry of data code 2053 (hypercholesterolaemia comorbidity) and/or value for data code 181 (cholesterol/HDL ratio) ≥5 mmol/L;
- Gout: entry of ICPC-1 code T92 (gout);
- Enrolment in care program for asthma: indication for enrolment in care program for asthma as per data codes 2406 (treating physician for asthma) and/or 3828 (enrolment in care program for asthma);
- Enrolment in care program for COPD: indication for enrolment in care program for COPD as per data codes 2407 (treating physician for COPD) and/or 3829 (enrolment in care program for COPD);
- Enrolment in care program for DM: Enrolment in care program for COPD: indication for enrolment in care program for DM as per data codes 2206 (treating physician for DM) and/or 3827 (enrolment in care program for DM);
- Enrolment in care program for any care program: indication for enrolment in one or more care programs of asthma, COPD or DM as defined above, or for indication for enrolment in care program for HF as per data codes 3016 (treating physician for HF) and/or 3833 (enrolment in care program for HF), or for indication for enrolment in care program for thyroid disease as per data codes 3040 (treating physician for thyroid disease) and/or 3835 (enrolment in care program for thyroid disease).

Supplementary Results

Comparison of patients with and without complete baseline CHARGE-AF data

Supplementary Table 1 shows a comparison between those free of AF at baseline with complete baseline CHARGE-AF data and those free of AF at baseline without complete baseline CHARGE-AF data (n=538,308). Five-year AF incidence was significantly lower among incomplete CHARGE-AF cases (2.10%, p<0.001). Patients with complete CHARGE-AF baseline data were significantly older and had significantly higher burden of cardiovascular comorbidities than patients with incomplete CHARGE-AF variables at baseline. The percentage of missing CHARGE-AF measurements varied from 69.3% (SBP) to 81.3% (height). Patients with at least 1 but not all 4 CHARGE-AF measurements recorded in the EHR in 2013 had a higher mean SBP, DBP and height, but lower weight, than patients with complete baseline CHARGE-AF measurements.

Additional CHARGE-AF validation analyses

In the stratified analyses on CHARGE-AF, discrimination was consistently higher in the lower risk groups (women, age <65 years and CHA_2DS_2-VASc <2), with highest C-statistic in the subgroup of women (0.751; 95%CI: 0.740-0.763). Calibration of CHARGE-AF was insufficient in all subgroups as assessed by the Nam-D'Agostino χ^2, and the calibration slope significantly deviated from 1 in all subgroups except in patients younger than 65 and in patients with CHA_2DS_2-VASc <2 (see Table 2 in main text).

Calibration plots for the stratified CHARGE-AF analyses were similar to that of the overall analysis, except in the subgroups age <65 years and CHA_2DS_2-VASc <2. In these lower risk strata, risk prediction was accurate for all deciles, without overestimation in the highest deciles seen in the other analyses (Supplementary Figure 4).

Supplementary Tables

Supplementary Table 1. Comparison between baseline characteristics of patients free of AF at baseline within all extracted Nivel-PCD participants and those with complete CHARGE-AF variables

	All (n = 649,783)	Complete CHARGE-AF variables at baseline (n = 111,475)	Incomplete CHARGE-AF variables at baseline (n = 538,308)	p-value for difference*
**Age, years**	58.2 ± 12.6	65.5 ± 11.4	56.7 ± 12.2	<0.001
**Female**	335,155 (51.6%)	58,549 (52.5%)	276,606 (51.38%)	<0.001
**AF during 5-year follow-up**	16,581 (2.55%)	5,264 (4.7%)	11,317 (2.10%)	<0.001
**SBP, mmHg**	137.9 ± 17.1 *450,044 (69.3%) missing*	137.3 ± 16.3	138.7 ± 18.1 *(from n = 88,264 non-missing)*	<0.001
**DBP, mmHg**	81.1 ± 10.8 *450,848 (69.4%) missing*	80.5 ± 10.5	81.9 ± 11.0 *(from n = 87,460 non-missing)*	<0.001
**Height, cm**	170.1 ± 9.9 *528,047 (81.3%) missing*	170.0 ± 9.9	171.2 ± 9.9 *(from n = 10,261 non-missing)*	<0.001
**Weight, kg**	82.2 ± 17.2 *516,993 (79.6%) missing*	82.5 ± 16.8	80.4 ± 18.6 *(from n = 21,315 non-missing)*	<0.001
**Antihypertensive medication**	188,122 (29.0%)	79,057 (70.9%)	109,065 (20.26%)	<0.001
**Hypertension**	177,537 (27.3%)	74,149 (66.5%)	103,388 (19.21%)	<0.001
**Diabetes mellitus**	72,467 (11.2%)	47,557 (42.7%)	24,910 (4.63%)	<0.001
**Heart failure**	12,753 (2.0%)	4,693 (4.2%)	8,060 (1.50%)	<0.001
**Myocardial infarction**	14,572 (2.2%)	5,404 (4.9%)	9,168 (1.70%)	<0.001
**Current smoking**	21,036 (3.2%)	15,774 (14.2%)	5,262 (0.98%)	<0.001
**Stroke**	19,380 (3.0%)	7,462 (6.7%)	11,918 (2.21%)	<0.001
**TIA**	8,630 (1.3%)	3,339 (3.0%)	5,291 (0.98%)	<0.001
**Pulmonary embolism**	2,208 (0.3%)	506 (0.5%)	1,702 (0.32%)	<0.001
**Angina pectoris**	28,328 (4.4%)	10,167 (9.1%)	18,161 (3.37%)	<0.001
**CHA_2DS_2-VASc**	1 (IQR 0-2)	3 (IQR 2-4)	1 (IQR 0-2)	<0.001
**CHA_2DS_2-VASc ≥2**	247,694 (38.1%)	88,538 (79.4%)	159,156 (29.6%)	<0.001
**Asthma**	57,929 (8.9%)	13,262 (11.9%)	44,667 (8.30%)	<0.001
**COPD**	35,252 (5.4%)	12,523 (11.2%)	22,729 (4.22%)	<0.001
**Atherosclerosis**	13,759 (2.1%)	6,367 (5.7%)	7,392 (1.37%)	<0.001
**Hypercholesterolaemia**	34,135 (5.3%)	19,427 (17.4%)	14,708 (2.73%)	<0.001
**Gout**	23,516 (3.6%)	7,639 (6.9%)	15,877 (2.95%)	<0.001
**Enrolled in care program for:**				
**Asthma**	4,374 (0.7%)	1,846 (1.7%)	2,528 (0.47%)	<0.001
**COPD**	8,572 (1.3%)	4,777 (4.3%)	3,795 (0.70%)	<0.001
**Diabetes mellitus**	38,969 (6.0%)	35,640 (32.0%)	3,329 (0.62%)	<0.001
**Any care program**	49,820 (7.7%)	40,468 (36.3%)	9,352 (1.74%)	<0.001

AF, atrial fibrillation; COPD, chronic obstructive pulmonary disorder; DBP, diastolic blood pressure; IQR, interquartile range; SBP, systolic blood pressure; TIA, transient ischaemic attack.

Data are number (percentage), mean ± standard deviation or median (IQR).

* Difference between those with and without complete baseline CHARGE-AF measurements

Supplementary Figures

Supplementary Figure 1. Study flowchart

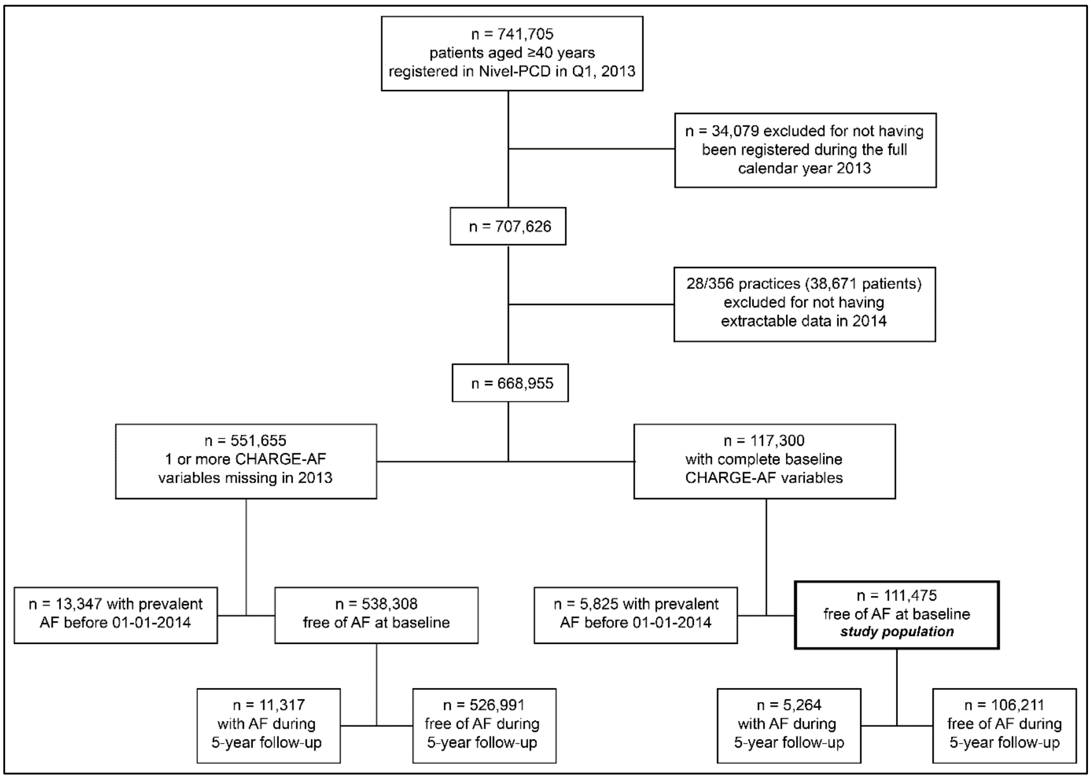

AF, atrial fibrillation; CHARGE-AF, Cohorts for Heart and Aging Research in Genomic Epidemiology model for AF; Nivel-PCD, Netherlands Institute for Health Services Research Primary Care Database; Q1, first quarter.

Supplementary Figure 2. Baseline CHARGE-AF risk distribution in the sample and relative contribution of CHARGE-AF risk factors to increments in baseline risk (n = 111,475 with complete baseline CHARGE-AF data)

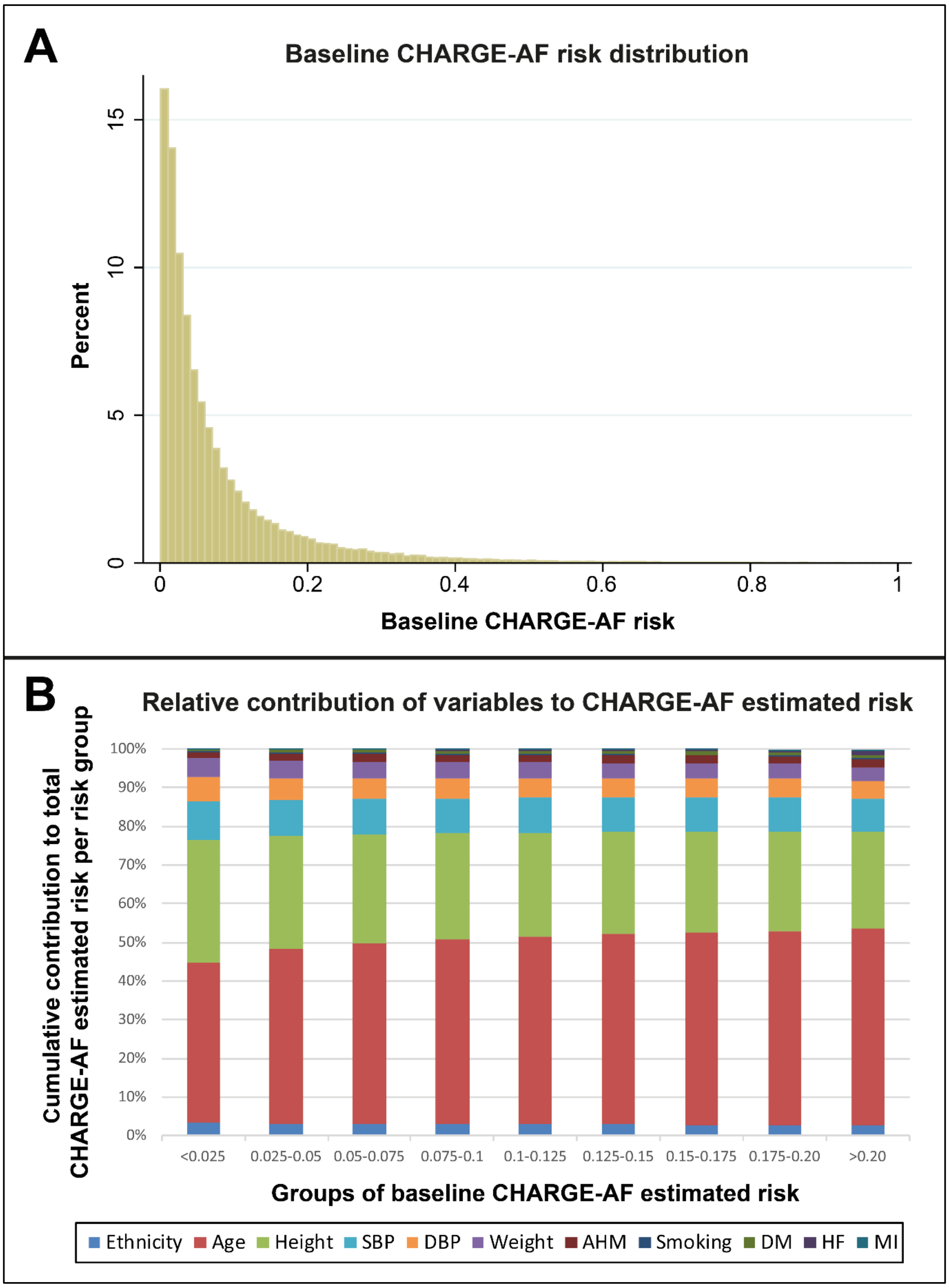

AHM, antihypertensive medication use; CHARGE-AF, Cohorts for Heart and Aging Research in Genomic Epidemiology model for AF; DBP, diastolic blood pressure; DM, diabetes mellitus; HF, heart failure; MI, myocardial infarction; SBP, systolic blood pressure.

Panel A, Baseline CHARGE-AF risk distribution; Panel B, Relative contribution of CHARGE-AF risk factors to mean baseline CHARGE-AF risk score in successive strata of increased CHARGE-AF risk. Since DBP has a negative coefficient in the CHARGE-AF formula, DBP is depicted as such in this graph.

Supplementary Figure 3. Cumulative AF incidence and number of practices included in the analysis during follow-up

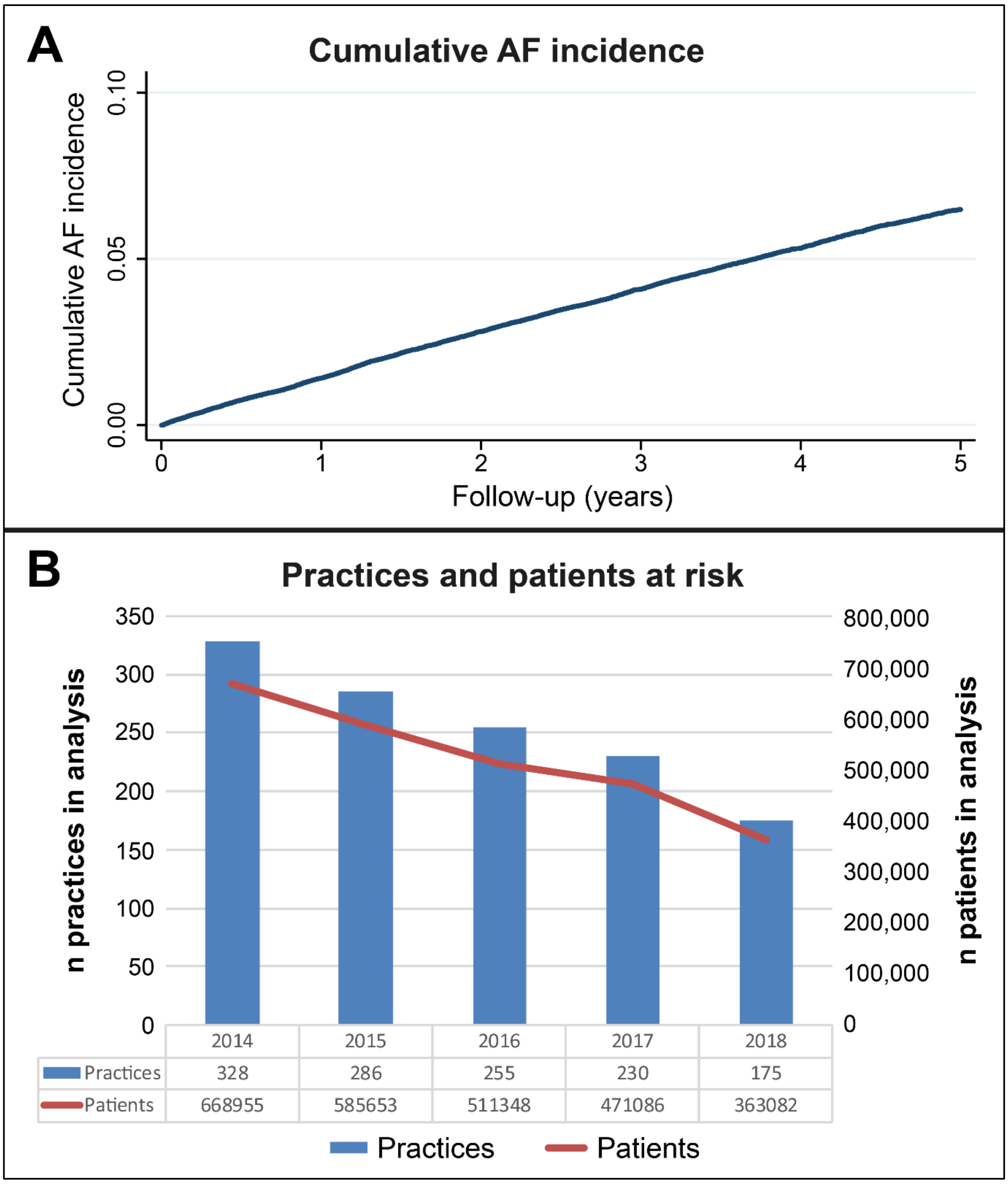

AF, atrial fibrillation; Nivel-PCD, Netherlands Institute for Health Services Research Primary Care Database. Panel A, Kaplan-Meier plot of cumulative AF incidence for all n=111,475 free of AF and complete CHARGE-AF data at baseline; Panel B, Number of Nivel-PCD practices (blue bars) and patients (red line) at risk during each Nivel-PCD extraction year.

Supplementary Figure 4. Calibration plots of CHARGE-AF in Nivel-PCD, stratified analyses

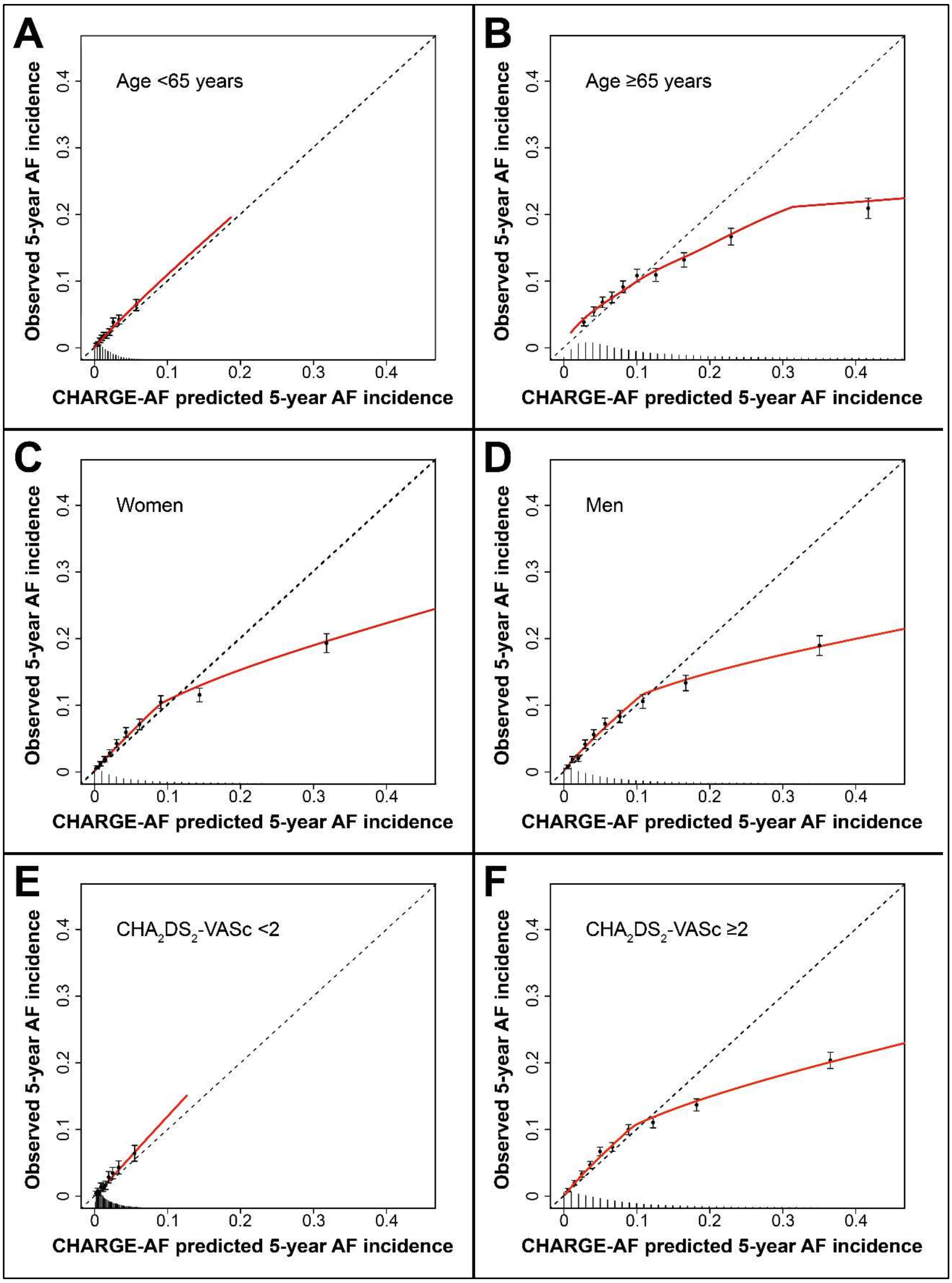

AF, atrial fibrillation; CHA_2DS_2-VASc, Congestive heart failure, Hypertension, Age, Diabetes and previous Stroke or Transient Ischaemic Attack, Vascular disease and female Sex category; CHARGE-AF, Cohorts for Heart and Aging Research in Genomic Epidemiology model for AF.

Panel A, analysis including all aged <65 years (n = 50,947); Panel B, analysis including all aged ≥65 years (n = 60,528); Panel C, analysis including all women (n = 58,549); Panel D, analysis including all men (n = 52,926); Panel E, analysis including all CHA_2DS_2-VASc <2 (n = 88,538); Panel F, analysis including all CHA_2DS_2-VASc ≥2 (n = 88,538).

The points indicate intersects of observed and expected for each decile of baseline CHARGE-AF risk, with brackets indicating the 95% confidence intervals of observed AF probability during 5-year follow-up in each decile. The red line indicates the trend for CHARGE-AF calibration in the sample. The spikes on the x axis indicate the distribution of AF-free survivors by CHARGE-AF risk.

References in Supplement

1. Nielen MMJ, Spronk I, Davids R, Korevaar JC, Poos R, Hoeymans N, et al. Estimating Morbidity Rates Based on Routine Electronic Health Records in Primary Care: Observational Study. JMIR Med Inform. 2019;7(3):e11929.
